# The draft genome sequence of *bla*_CTX-M-270_-carrying *Kluyvera ascorbata* isolated from human sputum

**DOI:** 10.1128/mra.00011-24

**Published:** 2024-03-05

**Authors:** Lina Liu, Yu Feng, Hongxia Wen, Zhiyong Zong

**Affiliations:** 1 Center for Pathogen Research, West China Hospital, Sichuan University, Chengdu, China; 2 Division of Infectious Diseases, State Key Laboratory of Biotherapy, Chengdu, China; 3 Center of Infectious Diseases, West China Hospital, Sichuan University, Chengdu, China; Rochester Institute of Technology, Rochester, New York, USA

**Keywords:** pathogen, CTX-M, *Kluyvera*, *Kluyvera ascorbata*

## Abstract

*Kluyvera ascorbata* is recognized as an opportunistic pathogen of the *Enterobacteriaceae* family but remains less studied. We report the draft genome of a *K. ascorbata* clinical strain recovered from human sputum, comprising approximately 5.18 million bases and harboring an intrinsic gene encoding the extended-spectrum β-lactamase CTX-M-270.

## ANNOUNCEMENT

*Kluyvera ascorbata* is a species of the *Enterobacteriaceae* (1) and can cause a variety of infections, including bacteremia, cholangitis, and urinary tract infections (2–4), some of which are life-threatening (5, 6). However, compared to many other *Enterobacteriaceae* species, *Kluyvera* remains less studied. Here, we present the draft genome of a *K. ascorbata* clinical strain.

Strain 141611 was isolated from the sputum of a patient with pulmonary infection at our hospital in 2023 as part of standard care.The sputum was inoculated onto blood and chocolate plates for 24-h incubation at 37°C. The strain was preliminarily identified as *K. ascorbata* using matrix-assisted laser desorption ionization time-of-flight mass spectrometry (Bruker, Billerica, MA, USA). Subsequently, the strain was cultured on Luria-Bertani agar at 37°C for 24 h, and a single colony was sub-cultured overnight in broth using the QIAamp DNA Blood Mini Kit (Qiagen, Hilden, Germany) for genomic DNA extraction following the instruction without modifications. Sequencing library was constructed using the NEBNext Ultra II kit (NEB, Ipswich, MA, USA) (7). Genome sequencing with a 150-bp paired-end protocol was performed on HiSeq X10 (Illumina, San Diego, CA, USA). Generated reads (*n* = 9,047,592, coverage, 262×) were processed with Cutadapt v4.0 (8) for trimming 10 bp from both ends and removing adapters and quality filtering (minimum quality, Q15; minimum length, 50 bp) using BBMap v39.01 (https://jgi.doe.gov/data-and-tools/software-tools/bbtools). Default parameters were used for all software unless otherwise specified. Reads were assembled into a draft genome of 5,180,442 bp in 81 contigs (G + C content, 54.13%) using SPAdes v3.15.3 (9). Strain 141611 was precisely identified as *K. ascorbata*, exhibiting a 98.28% average nucleotide identity with *K. ascorbata* ATCC 33433^T^ (accession no. JMPL00000000) determined using FastANI v1.33 (10). Three antimicrobial resistances, *bla*_CTX-M-270_, *fosA*, and *tet*(34), were identified using ResFinder (https://cge.cbs.dtu.dk/services/ResFinder). *Kluyvera* is known as the origin of *bla*_CTX-M_ genes (11). Susceptibility testing was performed using Vitek II (bioMérieux, Marcy-l'Étoile, France) using *Escherichia coli* ATCC25922 as the reference, and results were interpreted according to CLSI 2022 guidelines (12). Strain 141611 was resistant to piperacillin, cefuroxime, cephalothin, and cefazolin but was susceptible to amoxicillin/clavulanate, ticarcillin/clavulanate, ampicillin/sulbactam, piperacillin/tazobactam, ceftriaxone, cefepime, cefotaxime, ceftazidime, cefpodoxime, ertapenem, imipenem, meropenem, doripenem, amikacin, tobramycin, gentamicin, ciprofloxacin, levofloxacin, moxifloxacin, doxycycline, minocycline, tigecycline, tetracycline, and trimethoprim-sulfamethoxazole. The resistance to penicillin and the first- and second-generation cephalosporins may be explained by the presence of *bla*_CTX-M-270_.

**Fig 1:**
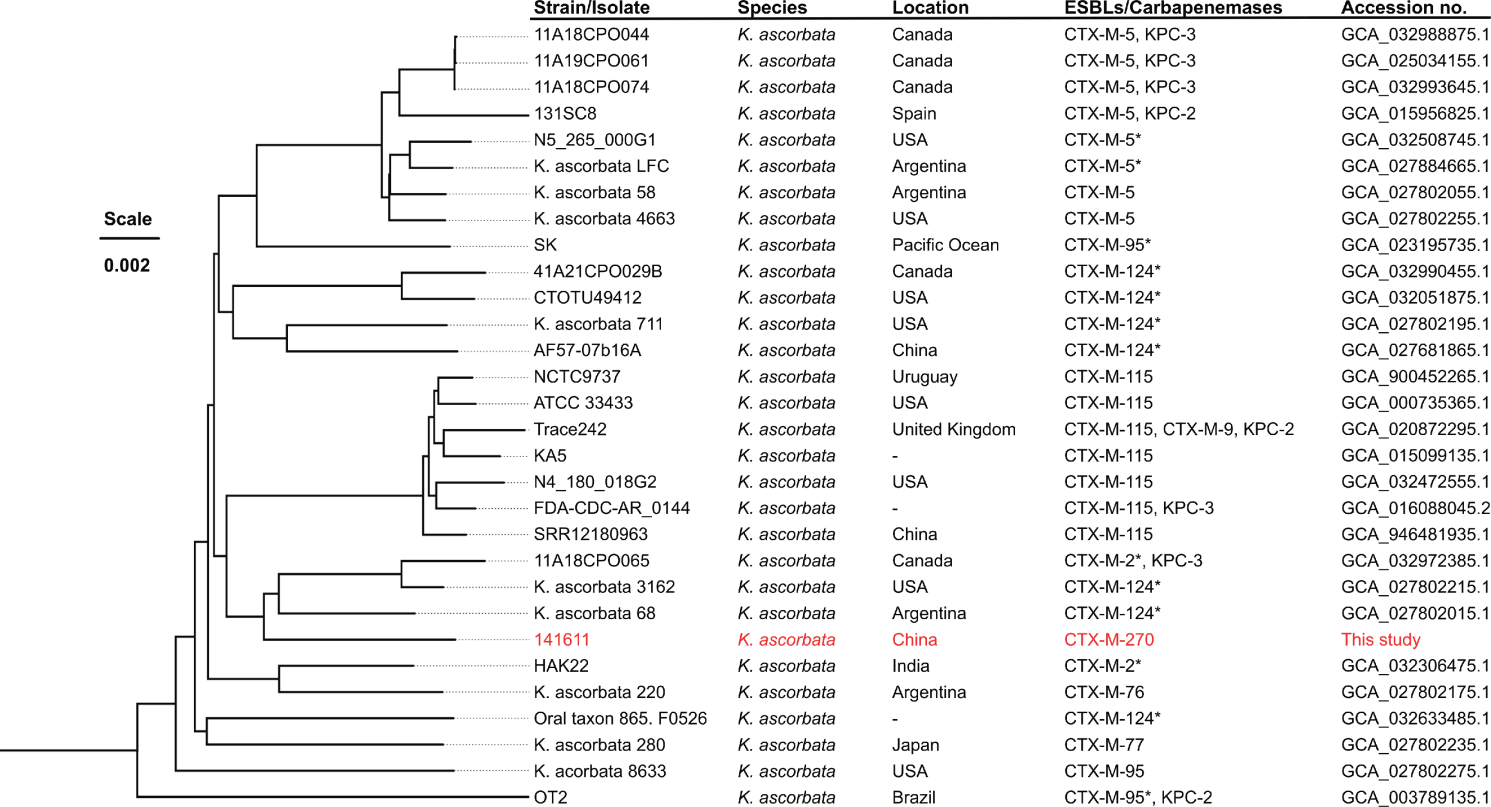
Phylogenomic tree of *K. ascorbata*. Strain 141611 is labeled in red. The asterisk indicates close to CTX-M variants. The tree was inferred from concatenated SNP alignments using IQ-Tree v2.2.3 under the GTR model applied with gamma distribution and 1,000 bootstrap tests.

For investigating the clonal relatedness of strain 141611 with other *K. ascorbata* strains, we retrieved all available *K. ascorbata* genome assemblies (*n* = 29) from NCBI and aligned with the complete chromosome of a *K. ascorbata* strain (SK, accession no. CP096201) using Snippy v4.6.0 (https://github.com/tseemann/snippy). Phylogenomic tree was inferred from a concatenated single nucleotide polymorphism (SNP) alignment using IQ-Tree v2.2.3 (13) using the GTR model with gamma distribution and 1,000 bootstrap tests. Strain 141611 had 33,351 SNPs with the most closely related strain 3162 (accession no. GCA_027802215) (Fig. 1), signifying its phylogenetical divergence from all other *K. ascorbata* strains with genomes available in NCBI.

## Data Availability

The whole-genome sequence of 141611 has been deposited in GenBank under the accession number JAXOAR000000000 and BioSample accession number SAMN38720711. The Illumina sequence reads have been deposited in the Sequence Read Archive (SRA) database under the accession number SRR27139443.
